# Market potential and opportunities for commercialization of traditional meat products in North East Hill Region of India

**DOI:** 10.14202/vetworld.2018.118-124

**Published:** 2018-02-05

**Authors:** G. Kadirvel, Bandita B. Banerjee, Surajit Meitei, S. Doley, Arnab Sen, M. Muthukumar

**Affiliations:** 1 Division of Animal Production, ICAR - Research Complex for NEH Region, Umiam, Meghalaya, India; 2 National Research Centre on Meat, Hyderabad - 500 092, Telangana, India

**Keywords:** commercialization, North East India, meat, products, property, traditional

## Abstract

The North Eastern (NE) India is renowned for its preference for animal-based food. This region is known for its traditional meat products. However, the popularity of these products remains confined to the specific community/location. The knowledge on the traditional preparation methods is generally passed across generations through practice and word of mouth. The traditional style of preparation and the specific ingredients added to each product makes them unique. In this review, an attempt has been made to identify the initiatives, opportunities, and market potential for commercialization of the traditional meat products. These unique features and properties of the traditional meat products have been highlighted. The commercialization of these products will enhance entrepreneurship development and ensure quality ethnic products to the consumer in the NE hill region of India.

## Introduction

India has a large number of ethnic groups, diverse tradition, culture, and varied food habits. A variety of food products of indigenous taste are being prepared and consumed in India, and these products vary from region to region and place to place. They are native in origin and termed as traditional or indigenous products. Traditional processing of various meat products with locally available specific ingredients has resulted in the development of products with unique sensory attributes. Based on the availability of raw materials, people have developed a taste to particular food products. Nowadays, these products are becoming popular and their demand for such products is increasing day-by-day.

The ethnic and tribal people of North East Hill (NEH) of India are confined to their traditional food habits with meat as an integral part. Diversity in tradition and culture among different communities in NEH region has resulted in a large variety of traditional meat products. The recent 19^th^ Livestock Census-2012 indicates that there is 38.21% increase in meat production from 1999-00 to 2012-13 against the national average of 29.21%. Similarly, the meat consumption pattern and expenditure are 2-3 folds higher when compared to national average [1], which underscore the importance of meat and meat products in the region. Varieties of products are being prepared from meat and fish with locally available vegetables, herbs, and spices. Among them, indigenously produced blood sausage, animal by-products with rice flour, maize, or fruits, dry meat powder with herbs, and special preparation from animal fats preserved in dry gourd or bamboo containers are important [2]. The shelf life of these products ranges from a few days to few months at room temperature. These products as well as methods of preservation vary with region and ethnic groups.

These products are usually confined to the community level, produced, and sold locally in small scale. The traditional food products are mostly prepared in individual households, which are not available in commercially in food stalls of the region. The knowledge on the preparation method is generally passed on from one generation to the other through practice and word of mouth. However, the native meat handlers are ignorant about the importance of maintaining hygiene and quality specifications during the preparation of such traditional products. These factors pose to be as constraints for commercialization in the current scenario. Nowadays, food consumption pattern has been changing due to increased income and livelihood of the people in the region, besides the high demand for ready to eat foods in the region. Further, a large population has been migrating to different parts of India for education, job, and business. Commercialization of the ethnic meat products and adequate marketing can cater the palette of this population and address to the huge demand for different meat products in various regions of the country. Moreover, as the meat-processing sector has relatively high-value addition to products, commercialization of traditional meat products can enhance the income of the tribes from traditional engagement in home-based food processing. With the increase in the population of working women in the urban sector, demand for processed products has increased to counter the time constraints of women for food preparation at home. Hence, commercialization of ethnic meat products can meet new consumer demands.

This review throws light on the growing importance of meat and meat products in the NE region and property of the traditional meat products, with expected market potential, scope, roadmap, and opportunities for commercialization of traditional meat products in the NEH region of India.

## Meat Production and Consumption Pattern of NEH Region of India

**Figure-1:**
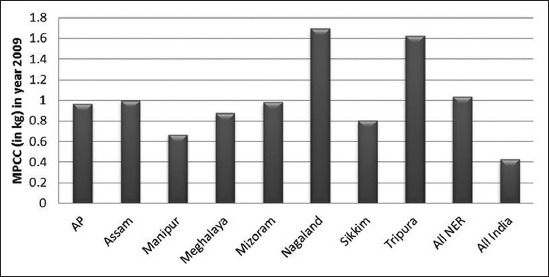
Meat consumption pattern in Northeast India.

**Figure-2:**
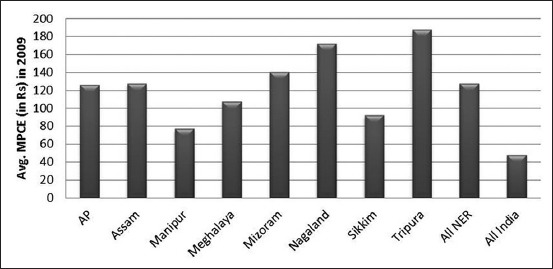
Expenditure on meat in Northeast India.

The dependence on livestock as an alternative source of income is significant in the agriculture sector in this region [3]. Meat is an important and common source of animal protein in the NEH states. Meat production in the recognized sector has also gone up by 38% in NEH region between the periods 1999-2000 and 2012-2013, which is higher than the increase in the national level of 29% (Table-1). All NEH states, except Arunachal Pradesh, have witnessed an increase in meat production. The consumption of pork is much higher (68.75%) in the region which reflects the significance of pork in the everyday diet of NE population. The importance of meat production and consumption in the region in the recent years seems to take a higher platform with few brands emerging in organized production and processing of meat, for example, Arohan Foods in Guwahati and Meat Treat in Shillong. In general, food consumption pattern is based on the income and purchased power and availability. On the contrary in NE India, the expenditure on meat is much higher as compared to that of rest of India, although the average annual per capita income is significantly lower than the national per capita income. It is evident from the expenditure estimate which shows that in the NE states around 16.5% and in mainland India only 7% is devoted to meat out of the total food expenditure [1]. The average monthly per consumer unit consumption and the average monthly per consumer unit expenditure pattern in NEH India are compared with the rest of India in Figures-1 and 2. The high demand for meat can be addressed with increasing livestock production and converting it into traditional products which can be commercialized and distributed in a large market crossing the boundaries in the region.

Prospective market for traditional meat products of NE India is summarized in Table-2.

**Table-1:** Estimated meat production in Northeastern India (000’tonnes).

States	1999-00	2012-13	% increase
Arunachal Pradesh	18.20	17.64	−3.17
Assam	25.13	36.60	31.34
Manipur	20.71	25.02	17.23
Meghalaya	21.49	38.52	44.21
Mizoram	9.38	12.08	22.35
Nagaland	20.36	70.64	71.18
Sikkim	2.00	3.00	33.33
Tripura	28.12	31.79	11.54
All NER	145.39	235.29	38.21
All India	4211.00	5948.17	29.21

Source: BAHS, 2014

**Table-2:** Prospective market for traditional meat products of Northeast India.

Traditional meat products	Present market	Market potential
DohJem, Tungrymbai	Meghalaya	All the States of Northeast India
Doh Snam	Meghalaya	All States of India and Export to South Asian Countries
Bongsha Rep, Vawksha Rep	Mizoram	All States of India and Export to Thailand, Burma, US, etc,
Vawksha Rep But, Bongsha Rep But	Mizoram	Packs to other States of Northeast India
Achar Doh Sniang	Meghalaya	All parts of the world
Khazing	Manipur	Meghalaya, Tripura, Arunachal Pradesh, etc.
Hentak	Manipur	Export to Asian and South Asian Countries
Anishi	Nagaland	Manipur, Meghalaya, Mizoram
Pork pickle	Nagaland	All States of India and Export to Asian and South Asian Countries.
Smoked Pork	Mizoram, Nagaland and Meghalaya	All States of India and Export to Asian and South Asian Countries

## Popular Traditional Meat Products in NEH Region of India

NE India harbors a wide variety of traditional meat products prepared by the natives of the region which reflects their social, cultural, spiritual, and ecological life. The process and techniques used for such preparations not only demonstrate their skill and creativity but also exhibit their capability to sustain the dynamics of life and ecosystem. There is a wide range of traditional meat products of NE India which is due to the use of different ingredients such as soybean, bamboo shoot, leafy mustard, sesame seeds, and rai in different combinations with different kinds of meat. The method of preparation varies on the basis of availability of materials, climate, culture, and overall knowledge of process and methods. Considering the diversities of the traditional meat products, the popular ones can be identified to explore its prospective potential in context to commercialization [4]. Earlier studies reporting on the traditional meat products of the region such as Manipur, Mizoram, and Nagaland focus on the uniqueness and popularity of the products.

In Meghalaya, appreciable number of such ethnic meat products can be identified. DohJem, as named in Khasi language, is a popular meat item among the natives of Meghalaya. It made from either pork or beef, primarily from offal such as the stomach, intestines, and clotted blood which is boiled and then fried with crushed sesame seeds and spices. Tungrymbai as locally known in Meghalaya is largely taken with everyday meals by the peasants of this state. It is a pork item prepared with fermented soybean and paste of sesame seeds cooked together with chopped ginger [5]. Pork pickle popularly known as Achar Doh Sniang by Khasi tribe, it is a favorite product for not only the people of Meghalaya but also for the entire tribe of NE India. The method of preparation may differ from one tribe to another, but the basic method is common. Boiled pork pieces are fried with spices which are pickled in bottles by dipping in mustard oil. Dohsnam, as named in Khasi, is basically pork sausage. It is a common dish in Mongolian-dominated Asian countries. However, the taste and style of preparation vary with place. Usually, the large intestine of the adult pig is used for casing of the product, and the content inside is mainly blood, sliced onion, smashed ginger, and smashed garlic with chilli and salt. Doh khleh is a delicious salad made of boiled pork, onion, and chilli. The dish mainly comprises of cuts of pork and animal soft parts (including intestines and brain) which is mixed gently with raw onion and chilli. Jadoh is a kind of rice preparation popular among the Khasi community of Meghalaya. It is red rice which is cooked with pieces of pork that gives an attractive look to the dish.

The traditional animal-based products of Manipur were reported by the studies of Devi and Kumar [6], Jeyaram *et al*. [7] which include primarily fish items such as Iromba, Champhu, Kangshoi, Hawaichar, Soibum, Ngaree, and Paknam. The use of various indigenous crop plants and meat of wild and domesticated animals for preparing boiled foods and fermented foods is a prevalent practice among the Naga tribe and Kuki tribe of Manipur. In a survey, 83 common and uncommon non-fermented traditional products of Sikkim are listed, of which many are animal-based [8]. The traditional knowledge used for preparing different fermented products of Sikkim including Langkargyong (sausage from beef/pork/yak meat), SukakoMasu (dried or smoked meat product from buffalo or chevon), Satchu (dried beef/pork/yak meat), and SukakoMaacha (smoked fish) has been documented [9]. Goyang, another popular product, is a preparation of beef or yak meat cooked with pre-processed leaves of a wild plant Maganesaag (*Cardamine macrophylla* Wild.) [10]. Chhwelaa is a typical boiled buffalo meat dish of the Newar community of Sikkim. During its preparation, a whole lump of meat is boiled and cut into pieces. It is mixed with a paste of spices, kneaded thoroughly, and then fried in oil. Fried fenugreek seeds and turmeric are garnished with meat. Falki is a special meat-based dish of the Gurung caste of the Nepali community in Sikkim [11].

Traditional meat products in Mizoram includes Sa-um (made from fermented pig fats), Sawhchiar (pork or chicken porridge), and Sarep (smoked meat). The Sarep is a broad category for smoked meat which includes domesticated as well as wild animals such as barking deer, (*Muntiacus vaginalis*), sambar deer (*Rusa unicolor*), wild boar (*Sus scrofa*), and macaque (*Macaca* sp.) [2]. Smoked beef is locally called by Mizo tribe as Bongsha Rep. When Bongsha Rep is cut into small pieces and boiled with salt, it is known as Bongsha Rep. However, Vawksha Rep is the local name for smoked pork in Mizoram. It is a very popular meat product among the pork-consuming population in the region. Vawksha Rep But is a boiled smoked pork preparation with mustard leaves and fermented soybean. Bong sung is another popular meat product, prepared from offal such as the spleen, heart, intestine, stomach, liver, and lung. Anishi is a product of pork or smoked pork which is prepared with edible *Colocasia* leaf. Anishi and Axone are the popular meat products in Nagaland. Axone (Aakhone) is prepared with meat and fermented soybean, and it is popular among the Sema Naga tribes [12]. These traditional products have significance on the unique substrates, preparation methods, starter cultures, etc. [13], which reflect the expertise of the native people in fermentation technique.

## Specialty/Properties of the Traditional Meat Products in NEH Region

Traditional meat preparations have their uniqueness imparting a cultural identity to the respective communities and tribes of NEH region of India. They are more palatable, nutritious, and better keeping quality at room temperature. The products can prove to be attractions for the health-conscious consumers as they are free from preservatives and artificial coloring agents. The techniques employed for processing and the special ingredients added are partially responsible for imparting the characteristic flavor to the products which make them unique. The most common techniques used in such traditional products are fermentation, smoking, and drying.

In the traditional fermentation process, acid is generated which is responsible for the enhancement of nutritional value and shelf life of the traditional meat products. The acid generators are primarily Gram-positive acidogenic lactic acid bacteria such as *Lactobacillus*, *Streptococcus, Pediococcus, Leuconostoc, Lactococcus*, and *Enterococcus* [13]. These can metabolize several *Saccharides* into lactic acid, alcohols, lipids, and some amino acids. Different bacteria have a different level of efficiency for metabolizing saccharides present either in the meat or ingredients added to the meat preparation such as bamboo shoot, sesame seed, and beans. In certain traditional products, these bacteria simultaneously reduce nitrate and nitrite to nitric oxide, the nitric oxide is combined with myoglobin protein of meat which gives cured color. The anaerobic glycolysis in the fermentation process produces lactic acid from glucose which reduces the pH of the products. The reduced pH range may be from 4.6 to 5 contributing to a sharp tangy taste and enhanced shelf life of the meat item [14].

The most popular technique used in traditional meat products is smoking. Smoked products are greatly prized by the food lovers in the region. The type and method of smoking vary from one tradition tribes to the other tribes in the region. Smoking adds flavor to the meat and also retards the growth of microbes such as bacteria, molds, and yeast on the surface of the meat. The flavor is added by the formation of innumerable antimicrobial and aroma compounds such as phenols and carbonyls and organic acids such as formic, acetic, propionic, butyric, and isobutyric [15]. These compounds help in coagulation of surface protein of the meat which inhibits microbial growth. The size of the smoked meat pieces influences the preservative effect of smoke because smoking is primarily a surface treatment. Hence, small-sized meat pieces when smoked are better than the large-sized ones. It adds antioxidative properties and enhances the organoleptic taste [16]. The duration, temperature, and source of smoke are responsible for the color and texture of the product.

The other common techniques used for the preparation of traditional meat products include frying, cooking, drying, and curing, besides special style which adds special features to the meat products. Drying reduces the water activity of the product aiding in longer duration of preservation. The process inhibits the growth of microorganisms as the level of available moisture for microbial growth is reduced in the product.

The traditional products play an important role as functional foods as well. The popular ingredients added in the products such as fermented bamboo shoot, soybean, and sesame seeds have immense functional properties which increase the nutritional content of the traditional meat products as well as enhance the health benefits from the product. For example, fermented soybean imparts a characteristic flavor to the products and increases the nutritional value. Tungrymbai, the soybean-based fermented food of Meghalaya, is one such example which serves as a cheap source of protein in the diet. The protein content in Tungrumbai has been reported to be 45.9 g% on a dry weight basis and fat, fiber, and ash to be 30.2, 12.8, and 5.5 g%, respectively [17]. Fermented soybean is also reported to have antihypertensive properties. It plays an important role to prevent cardiovascular diseases as well [18].

Another popular ingredient in traditional products is bamboo shoot. Bamboo shoot is fermented in various ways to be added to the meat product. It is, especially, popular in Manipur. Different species of bamboo shoots are used, the common among which are *Bambusa arundinacea*, *Bambusa pallida*, and *Bambusa nana* [6]. Bamboo shoot is considered as a healthy food because of rich contents of proteins, carbohydrates, vitamins, fibers, and minerals and very low fat. It helps in the function of pituitary and thyroid glands. It reduces fats and cholesterol levels in blood [19].

Sesame seeds are one of the common ingredients in many traditional meat products which contain nutritionally rich important vitamins and minerals such as niacin, thiamin, Ca, P, and Fe. These are also rich in functional properties as they contain nutraceutical compounds such as tocopherols and phenols which have antioxidant properties helping in reducing blood pressure, cholesterol, degeneration of blood vessels, and chronic diseases [20].

The variety of spices such as turmeric, ginger, black pepper, chili, cardamom, garlic, cinnamon, and mustard used in the traditional meat cuisines also affect the appeal and nutritional content of the products. Each spice has its own array of health benefits. The curcuminoids of turmeric are naturally occurring antioxidants that provide innumerable health benefits such as anti-inflammatory and anticancer action. The anticancer activity of turmeric is due to the presence of the carbonyl group in curcumin present in turmeric [20]. Ginger is beneficial in cold and cough due to the presence of gingerols and shogaols which exhibit antimicrobial activity [21]. The extract of ginger may minimize the problem of immune-depressed individuals, such as patients with chronic diseases or HIV-positive patients [22]. Black pepper and chili are a rich source of Vitamin C for the body.

Innumerable benefits of such kinds can be obtained from spices which are added to the traditional products. It enhances the functional property of the product. In addition to the enhancement of flavor and nutritional value of the dishes, the spices contribute to the stimulation of lactic acid bacteria due to their antioxidant properties. They also have an antimicrobial effect on certain kinds of microorganisms. The synergistic effect of spices in fermentation is due to the presence of manganese in spices such as black pepper, mustard, garlic, nutmeg, cinnamon, and mace [23]. Manganese has been cited as a growth factor for cultures and an inhibitory factor for *S. aureus* and *L. monocytogenes* [24,25]. The role of spices in fermentation depends on the nature and quality of the spice and the type and source of the spice and the microorganisms involved in the process.

## Constraints in the Traditional Meat Products

In spite of possessing innumerable advantages, the traditional meat products have a number of shortcomings which act as constraints for commercialization at a broader level. These include:

- Lack of quality control and hygiene by meat handlers/producer
- Lack of standardization uniform - protocol which is acceptable taste for larger population
- Lack of adequate logistics for scaling up to large production
- Lack of knowledge on proper packaging materials and transport system
- Lack of institutional support mechanism
- Lack of branding and trademark in manufactured products
- Lack of market networking
- Lack of availability and accessibility of meat processing equipment and technical know-how on handling
- Lack of training for skill development of entrepreneurs interested in setting up processing units
- Lack of managerial and marketing skills for entrepreneurship development.

## Scope for Commercialization

A step forward can be taken for commercialization of traditional meat products by taking active initiatives on certain aspects such as screening and assessing the outstanding foods from the existing platter, refining them through subsequent secondary or tertiary processing and value addition, setting up location-specific industries and enterprises, and facilitating marketing network through cooperative societies, private sectors, and self-help groups. Such an initiative will require the formal involvement of private sectors, scientific institutions, and financial support from government, NGOs, and banks. However, before taking such initiatives, certain criteria require substantial input at the ground level for successful commercialization.

### Quality control and hygiene

The primary factor for successful commercialization of the traditional meat products is hygiene and quality control. Following of quality standards is mandatory on the production site. The production of the products should be hygienically done keeping in consideration the Hazard Analysis Critical Control Point and good manufacturing practices guidelines. The nutritive value of each traditional product is needed to analyze and maintain the quality.

### Standardization and documentation

A step forward for commercializing the products needs a uniform optimized technique for each product which can be documented for reference. These standardized protocols can be followed on a global scale helping the indigenous products to go global. Assessment self-life at different temperature for different products needs to be evaluated.

### Scaling-up

Extending the traditional meat products from local consumption to commercialization requires research trail on the technicalities of mass production of the products, bulk packaging, and temperature maintenance during transport and conditions of the distri­bution channel. On the basis of the trail, the logistics should be worked out for scaling-up production from small scale to a large scale.

### Packaging and transport

Hygienic packing is the key to commercialization of traditional products. To withstand the different levels of marketing stages necessitates proper packaging and transport. The package needs to be customized depending on the particular product, duration of storage, and transport. Road transportation is an important mode of travel in the hilly areas and road connectivity and related infrastructures need to be enhanced for sustainable marketing in the region.

### Cold chain

Cold chain is a climate-controlled supply chain consisting of storage and distribution activities, which maintain the traditional products at recommended ambient temperature range. The use of mobile refrigerators and freezers, namely, truck, car, van, and containers for transportation of the products which ensure shelf life, quality, and salability of the products.

### Skill and Entrepreneurship development

Training and skill development of the entrepreneurs in meat processing and scientific meat preparation methods, demonstration, and production of different meat products is required. Hand holding for the establishment of a small-scale mini-meat processing unit in respective locations and providing technical backup and input supply to promote entrepreneurship are required.

### Awareness of the Food Safety Standards Authority of India (FSSAI)

The FSSAI is the licensing authority for any food industry in India. Entrepreneurs need to be aware of the existence of FSSAI and set up the processing unit as per norms of FSSAI.

### Branding and trademark

To make an identity in the competitive market, branding of the products is very essential. The brands when given a particular trademark, it helps in image development of the brand in the minds of the consumer helping in deeper penetration into the market.

### Market networking

Entrepreneurs can establish linkage with marketing agencies to develop a network which will prove to be beneficial in market expansion and establish an adequate balance between supply and demand.

## Model for the Institutional Support Mechanism and Entrepreneurship Development in Traditional Meat Products in the Region

**Figure-3:**
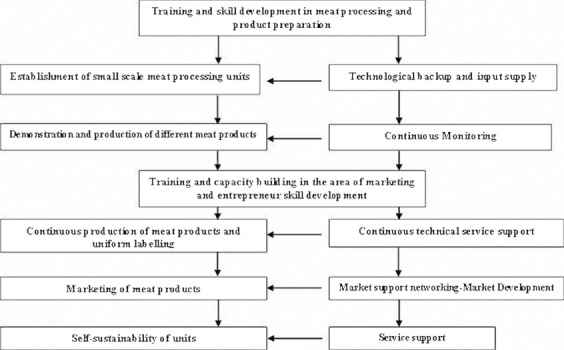
Model for the Institutional Support Mechanism and Entrepreneurship Development in traditional meat products in the region.

Commercialization of traditional meat products successfully also requires the intervention of certain institutional support mechanisms as shown in Figure-3.

### Export potential of NE India in traditional meat products

The traditional meat products have a potential market outside the region, besides the local market. These products can cater the palette of the South Asian countries since the taste is similar to their cuisine. A number of traditional meat products can be identified which have a promising demand in the ASEAN market. To support such kinds of initiatives from NE, a NE Cell has been set up in the Department of Commerce. The region has the support of an Export Development Fund (EDF) for promoting exports from the region. Activities which have a linkage with exports from the region are eligible for assistance from the EDF. The Agricultural and Processed Food Products Export Development Authority works as a nodal agency for sanctioning projects received under the EDF for NER [26]. Further, the NEH region of India share many international borders which could enhance the export potential of the products.

### Support by Government of India

The Ministry of Food Processing Industries, Government of India, supports initiatives in meat processing by providing integrated cold chain and preservation infrastructure facilities without any break from the farm gate to the consumer. It covers pre-cooling facilities at production sites, reefer vans, mobile cooling units as well as value addition centers which includes infrastructural facilities like Processing/Multi-line Processing/Collection centers. Small Farmer Agri-Business Consortium also provides assistance to setting up of cold storage by giving subsidies.

## Conclusion

The richness of NE India in traditional cuisine of animal origin is immense. When showcased in a common platform, the extensive potential of the products for commercialization becomes evident. Commercialization of these indigenous meat preparations will help in converting the local market into a global industry which will generate employment and self-sustainability in the region. It is important to orient their export policies to other border states. The commercialization of these products will enhance entrepreneurship development and ensure quality ethnic products to the consumer in the NEH region of India.

## Authors’ Contributions

GK and BBB: Conceived the idea, analyzed, and drafted the manuscript, SM: Survey on the traditional products, and SD, AS, and MM: Read the manuscript and corrected the final manuscript. All authors read and approved the final manuscript.
